# Exosomal proteomics reveals fatty acid metabolism linked to gefitinib resistance in non-small cell lung cancer

**DOI:** 10.1007/s10565-025-10121-8

**Published:** 2025-12-19

**Authors:** Yuanyuan Zhang, Wenjie Zhu, Jiang Zhu, Rui Hu, Yunhuang Yang

**Affiliations:** 1 State Key Laboratory of Magnetic Resonance Spectroscopy and Imaging, Key Laboratory of Magnetic Resonance in Biological Systems, National Center for Magnetic Resonance in Wuhan, Wuhan Institute of Physics and Mathematics. Innovation Academy for Precision Measurement Science and Technology, Chinese Academy of Sciences - College of Life Science and Technology, Huazhong University of Science and Technology, West No.30 Xiao Hong Shan, Wuhan, 430074 China; 2 University of Chinese Academy of Sciences, Beijing, 10049 China

**Keywords:** NSCLC, Exosomes, Gefitinib resistance, Proteomics, Fatty acid metabolism

## Abstract

Exosomes play a crucial role in the transmission of drug resistance in tumors. However, the mechanism of exosomes-mediated transmission in non-small cell lung cancer (NSCLC) under gefitinib treatment remains limited. In this work, we demonstrated that exosomes derived from HCC827/GR cells (drug-resistant) enhanced the survivability of HCC827 cells (drug-sensitive) under treatment with gefitinib. A total of 157 shared upregulated proteins between exosomes and their parent cells were identified in the comparison of the gefitinib-resistant groups versus the gefitinib-sensitive groups. Notably, 69 of these shared proteins are enzymes, and many of them were enriched in pathways related to fatty acid metabolism. Among these enzymes involved in fatty acid metabolism, ACC1 exhibited the highest fold change in upregulated expression in both drug-resistant groups (exosomes and cells). Moreover, the expression of ACC1 was upregulated in gefitinib-sensitive cells after uptake of exosomes from gefitinib-resistant cells. The role of ACC1 in enhancing the survival of HCC827/GR cells under gefitinib treatment was demonstrated using an inhibitor and siRNA-mediated knockdown. Specifically, the upregulated ACC1 stabilized fatty acid oxidation and reactive oxygen species levels in HCC827/GR cells, thereby maintaining cellular metabolic homeostasis. Collectively, this work reveals the transmission of drug resistance in NSCLC via exosomes that carry the ACC1 protein.

**Graphical Abstract:**

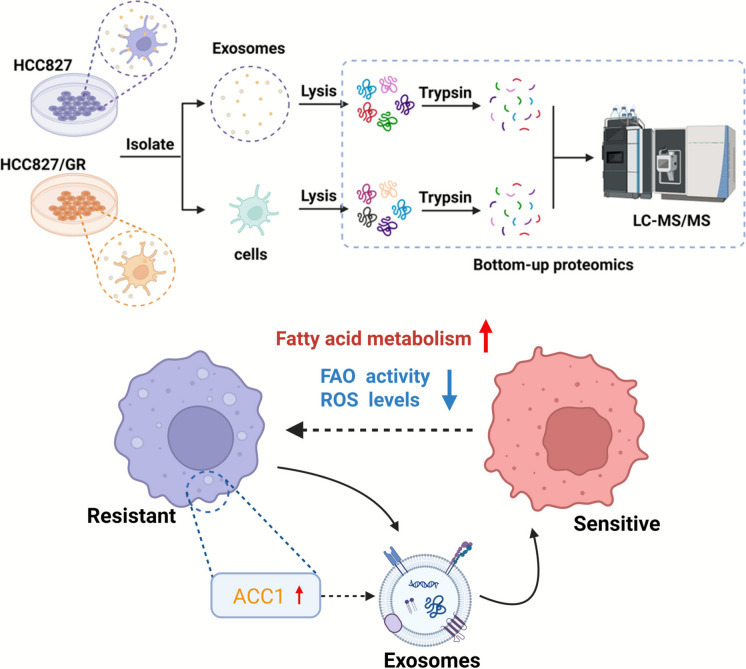

**Supplementary Information:**

The online version contains supplementary material available at 10.1007/s10565-025-10121-8.

## Introduction

Non-small cell lung cancer (NSCLC) continues to be one of the most prevalent and lethal malignancies worldwide, contributing significantly to cancer incidence and mortality (Bray et al. 2018; Duma et al. 2019). Despite the initial efficacy of targeted therapies such as gefitinib in patients with EGFR mutations, the emergence of resistance significantly hampers long-term treatment outcomes, with the T790M mutation accounting for 50% to 60% of resistant cases (Ciardiello et al. 2024; Singh et al. 2023; Zhu et al. 2019). Recent studies have shown that the tumor microenvironment (TME) plays a crucial role in developing resistance (Altorki et al. 2019; Klein 2012; Quail and Joyce 2013). The interactions between tumor cells and surrounding components, including the extracellular matrix, immune cells, and blood vessels, can profoundly affect the metabolic state of tumor cells and drive mechanisms of resistance. For instance, hypoxia and nutrient deficiency within the tumor microenvironment can trigger metabolic reprogramming in cancer cells, enabling them to adapt to drug treatment and ultimately develop resistance (Luo and Wang 2019). Understanding the complex interplay between resistance, the tumor microenvironment, and metabolism is vital for elucidating resistance mechanisms and developing novel therapeutic strategies.

Exosomes, nanoscale extracellular vesicles actively secreted by cells, are critical mediators of cell-to-cell signaling and metabolic homeostasis within the tumor microenvironment (Colombo et al. 2014; S et al. 2013; Théry 2011). These exosomes carry information from tumor cells and influence intercellular interactions and metabolic pathways within the TME (Deepak et al. 2020; Li et al. 2021). Tumor cells release exosomes that may alter the metabolic states of adjacent cells, thereby promoting tumor progression and the development of resistance (Liu et al. 2020; Mashouri et al. 2019). Furthermore, the metabolically relevant components within exosomes can serve as signaling molecules, influencing the function of immune cells, tumor cell proliferation, and the behavior of other cell types (Isaac et al. 2021; Ye et al. 2023). By studying the role of exosomes in the tumor microenvironment, we can gain a more comprehensive understanding of resistance mechanisms, particularly in the context of metabolic reprogramming.

The rapid advancement of proteomics provides a powerful tool for comprehensive analysis of lung cancer cell metabolism and resistance mechanisms (Wang et al. 2022; Wu et al. 2023). Current research suggests that significant shifts in the proteome of tumor cells often accompany the development of resistance (Fu et al. 2020; Wu et al. 2023). These changes reflect cellular adaptations to external drug pressures, suggesting metabolic reprogramming. Some proteins have been frequently verified as being associated with changes in the activity of key signaling pathways, such as the AMPK and PI3K-AKT pathways, which play pivotal roles in regulating cellular metabolism and survival (Herzig and Shaw 2018; Hoxhaj and Manning 2020). By systematically analyzing the proteome of gefitinib-resistant cells and integrating the molecular characteristics of exosomes, it is possible to effectively identify biomarkers and regulatory networks associated with resistance.

HCC827/GR is a gefitinib-resistant derivative of the parental human NSCLC cell line HCC827, which harbors an activating EGFR exon 19 deletion mutation. While HCC827 cells are highly sensitive to EGFR tyrosine kinase inhibitors (EGFR-TKIs) such as gefitinib, HCC827/GR cells have been established through chronic exposure to increasing concentrations of gefitinib, thereby acquiring stable resistance to the drug. This model recapitulates clinically relevant mechanisms of acquired resistance and is widely utilized in research on NSCLC drug resistance. In this study, we employed cellular and exosomal proteomics of HCC827 and HCC827/GR cells to explore how exosomes modulate the tumor microenvironment and influence tumor resistance. After bioinformatics analysis and functional validation, ACC1, a limited enzyme in fatty acid metabolism, exhibited significantly upregulated expression in resistant groups (both exosomes and cells) compared to sensitive groups, and stabilized fatty acid oxidation (FAO) and reactive oxygen species (ROS) levels, ultimately enhancing the survival capacity of NSCLC cells under treatment with gefitinib.

## Materials and methods

### Cell culture and transfection

HCC827 and HCC827/GR cell lines (CTCC-001–0364 and CTCC-GR-001; MeisenCTCC, China) were maintained in DMEM supplemented with 10% fetal bovine serum (FBS), penicillin (100 U/mL), and streptomycin (100 μg/mL) under 5% CO₂ at 37 °C. To sustain drug resistance, HCC827/GR cultures were supplemented with 8 μM gefitinib. Cells were transfected with ACACA-specific small interfering RNA (siRNA) (GenePharma, China) oligonucleotides using siRNA Mate Plus (GenePharma, China) at a final concentration of 25 nM.

### Viability and proliferation assays

Cells (3 × 10^3^/well) were seeded in 96-well plates, allowed to adhere overnight, and then exposed to a gradient of drug concentrations for 48 h. Viability was quantified using the CCK-8 assay (Beyotime, China), with absorbance measured at 450 nm after 2 h incubation.

### Exosome isolation and characterization

Upon reaching 80–90% confluency, the cells were washed with phosphate-buffered saline (PBS) and serum-starved for 48 h. Conditioned media underwent sequential centrifugation (2,000 × g, 30 min; 10,000 × g, 30 min) to remove debris and large vesicles. Exosomes were enriched using a total exosome isolation kit (Yeasen, China) via overnight incubation at 4 °C, pelleted (10,000 × g, 60 min), and resuspended in PBS. Protein content was determined by BCA assay (Thermo Scientific, USA). For functional studies, 100 μg of exosomal protein (equivalent to 1 × 10^7^ producer cells) was applied to 1 × 10^5^ recipient cells.

### Exosome validation

For the transmission electron microscope (TEM), exosome suspensions (10–20 μL) were adsorbed onto carbon-coated copper grids, air-dried, and negatively stained with 2% phosphotungstic acid (Macklin, China). Imaging was performed using TEM (Hitachi, Japan). Particle size and concentration were analyzed via nanoparticle tracking (ZetaView 8.04.02, Particle Metrix, Germany) across 11 measurement positions.

### Fluorescence microscopy analysis of exosome internalization

HCC827 or HCC827/GR cells were incubated with medium containing 5 μmol/L DiI (red) (Beyotime Biotechnology) at 37 °C for 20 min and then washed three times with PBS. We added DiO (Beyotime Biotechnology) to a 100 μg exosome suspension at 5 μmol/L and incubated for 20 min. Then, we washed the suspension with Exosome Spin Columns (Invitrogen) to remove excess dye. DiO-labelled exosomes were incubated with DiI-labelled cells for 24 h, and images of exosome uptake were obtained by fluorescent microscopy (Olympus).

### Animal experiments

Animal experiments were performed in compliance with relevant institutional guidelines, following approval from our Institutional Animal Care and Use Committee (IACUC). Male athymic BALB/c nude mice, aged 4 weeks and maintained under specific pathogen-free (SPF) conditions, were used in this study. All mice received subcutaneous transplantation of HCC827 cells into the right flank region, with 5 × 10^6^ HCC827 cells suspended in 100 μL of PBS administered per mouse. Once tumor volumes reached 100–150 mm^3^ (after approximately 14 days), the mice were randomly assigned to 3 groups: control group, HCC827-derived exosome treatment group, and HCC827/GR-derived exosome treatment group. All groups were administered gefitinib (5 mg/kg) via oral gavage once daily for 12 consecutive days. For the groups treated with HCC827-derived or HCC827/GR-derived exosomes, 100 μg of exosomes were injected intratumorally every 2 days. Tumor size was evaluated every 3 days using a digital caliper, with measurements of tumor length (L) and width (W) recorded. Tumor volume was calculated using the formula: V = L × W^2^/2. After 10 days, all mice were euthanized by cervical dislocation for subsequent analysis.

### Proteomic sample preparation

#### Protein extraction

Cells were lysed in RIPA (Beyotime, China) buffer containing 1 mM PMSF (Beyotime, China) on ice. Lysates were sonicated, cleared by centrifugation (12,000 × g, 10 min), and quantified using the BCA method. Exosome pellets were solubilized in 8 M urea (Merck, Germany).

#### Protein digestion

Proteins (50 μg) were acetone-precipitated (−20 °C, overnight), washed, and denatured in 8 M urea/10 mM dithiothreitol (DTT) (37 °C, 2.5 h). Alkylation was performed with 40 mM iodoacetamide (30 min, dark), followed by tryptic digestion (1:50 w/w, Promega, USA) in 50 mM NH₄HCO₃ (37 °C, overnight). Digests were acidified with 10% trifluoroacetic acid (TFA) for desalting.

#### Peptide desalting

C18 StageTips were activated with 80% acetonitrile/0.1% TFA and equilibrated with 0.1% TFA. Peptides were loaded, washed, and eluted with 80% acetonitrile/0.1% formic acid. Eluates were vacuum-dried and reconstituted in 0.1% formic acid (FA) for liquid chromatography-tandem mass spectrometry (LC–MS/MS).

### DIA data acquisition on orbitrap exploris™ 480

Peptides were separated on a C18 column (75 μm × 250 mm, Thermo Scientific, USA) using a 65-min gradient (eluent B, 0.1% FA in 80% acetonitrile) at 300 nL/min. The gradient was set as follows: 0–2 min, 4%–8% B; 2–37 min, 8%–25% B; 37–56 min, 25%–40% B; 56–65 min, 99% B. DIA data were acquired on an Orbitrap Exploris 480 (Thermo Scientific) with MS1 (120,000 resolution, 300% AGC) and MS2 (30,000 resolution, 2000% AGC) scans (400–1200 m/z). Maximum injection time for MS2 was 40 ms. HCD fragmentation was performed at a collision energy of 30%.

### Data analysis

Raw data were processed via DIA-NN (v1.9.2) against the UniProt Human database (October 2024 release). Search parameters included trypsin digestion, carbamidomethylation (fixed), methionine oxidation (variable), and 1% FDR thresholds. The result was exported to R (v 4.3.2) for statistical analysis. Statistical comparisons utilized unpaired t-tests, with data presented as mean ± standard error of the mean (SEM). Differentially expressed proteins (fold change > 1.5 or < 0.67, p < 0.05) were subjected to Gene Ontology (GO) and Kyoto Encyclopedia of Genes and Genomes (KEGG) pathway enrichment using DAVID (https://davidbioinformatics.nih.gov/) and KOBAS (http://bioinfo.org/kobas/), respectively.

### Western blot

Approximately 20 μg of cell lysates were separated using 8–16% SDS PAGE and transferred onto polyvinylidene fluoride membrane (PVDF). Membranes were blocked with QuickBlock™ (Beyotime) followed by incubation with primary antibody at 4 °C overnight and incubation with the secondary antibodies for 1 h at room temperature. An enhanced chemiluminescent (Epizyme) chromogenic substrate was used to visualize the bands. Antibodies for TSG101(1:15000), CD9(1:15000), ACC1 (1:3000), Tublin-β (1:25000) were purchased from Proteintech.

### FAO rate measurement

The FAO rate was evaluated using a colorimetric assay kit (Elabscience, E-BC-K784-M) according to the manufacturer’s instructions. Briefly, after centrifugation of 1 × 10^6 cells, the supernatant was discarded, and 200 μL of normal saline was added each time, followed by three washes. After washing, 200 μL of normal saline homogenate was added, and the sample was centrifuged at 10,000 × g for 15 min at 4 °C. The supernatant was placed on ice for measurement, and a portion of the supernatant was retained for determination of protein concentration. The absorbance at 450 nm was measured after the protein concentration was standardized using the Pierce BCA Protein Assay Kit (Thermo Fisher Scientific). The amount of enzyme that hydrolyzed the substrate to produce NADH at 37℃ by 1 g protein/min was defined as 1 unit by the following formula: FAO capacity (U/gprot) = (ΔA450-B)/a ÷ T × 1,000/Cpr (ΔA450: OD sample-OD control). Teacher: Response time, 30 min. Cpr: concentration of protein in the sample).

### Detection of ROS level

Cells were seeded in confocal plates for culture, and after adherent cells were treated with 8 μM gefitinib for 24 h, ROS assays were performed. Intracellular ROS levels were quantified using 2',7'-dichlorodihydrofluorescein diacetate (DCFH-DA; Yeasen, China). After staining, the cells were transferred to a confocal laser scanning microscope for image acquisition. All acquired images were analyzed using ImageJ software.

## Results

### Exosomes derived from HCC827/GR increased the gefitinib resistance of HCC827 *in vivo *and vitro

**Fig. 1:**
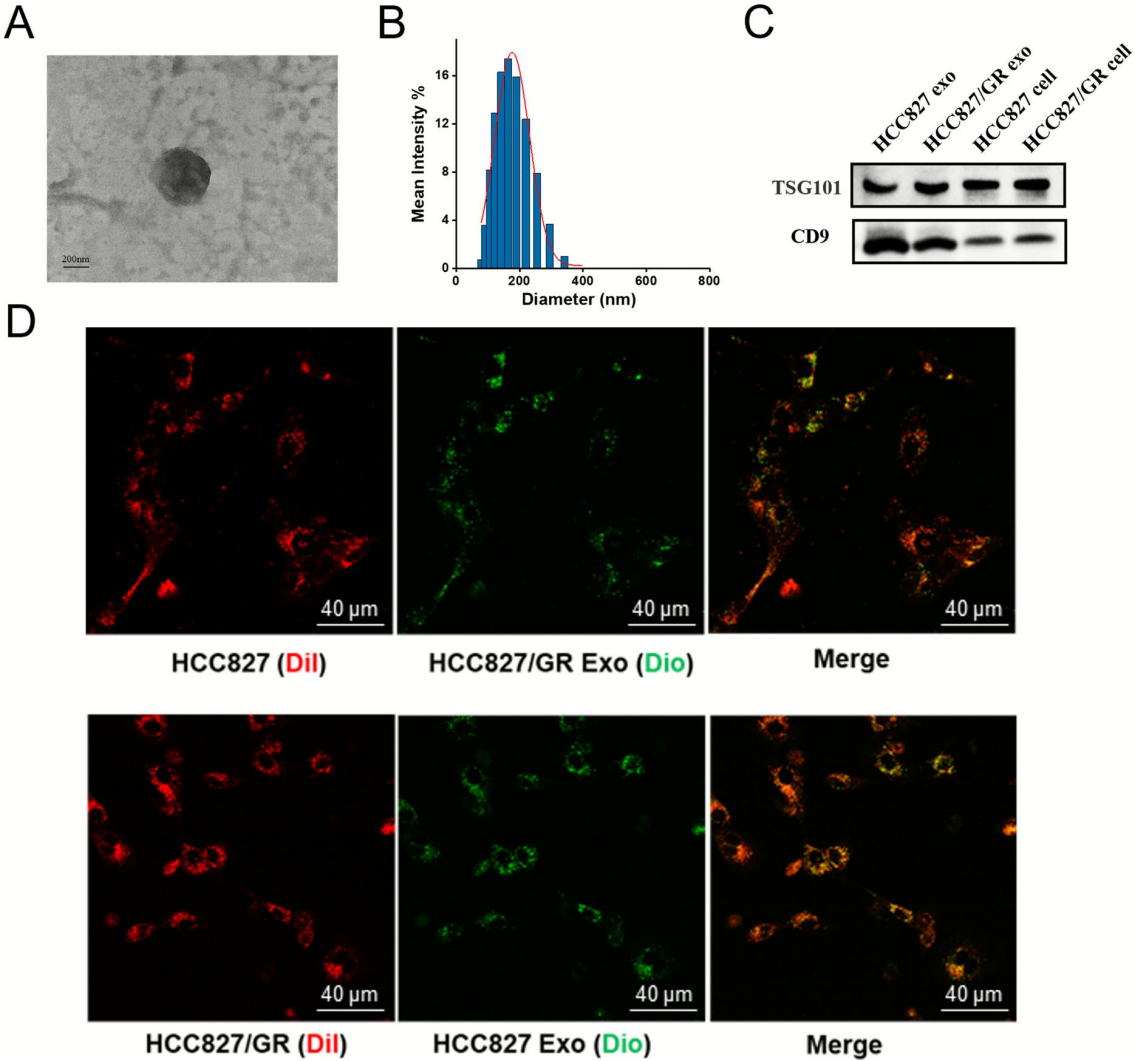
Exosome characterization and HCC827/GR cell-derived exosomes induce gefitinib resistance in HCC827 cell in vitro. (**A**) Representative TEM images of exosomes isolated from the supernatants of NSCLC cells. Scale bar, 200 nm. (**B**) NTA analysis of the size distribution and number of exosomes. (**C**) Western blot analysis showed that exosomal marker TSG101and CD63 was expressed in exosomes. (**D**) Representative fluorescent and merge images about fluorescence microscopy observation of DiI-labelled (red) HCC827 and HCC827/GR cells 24 h after incubated with DiO-labelled (green) exosomes. Scale bar, 40 μm

To evaluate the role of NSCLC-derived exosomes in drug resistance modulation, exosomes were isolated from conditioned media via polymer-based precipitation and validated through TEM and nanoparticle tracking analysis (NTA). Both HCC827 and HCC827/GR-derived exosomes displayed comparable morphological and biophysical characteristics, including a typical cup-shaped ultrastructure (Fig. 1A) and a size distribution profile (80–150 nm) consistent with exosomal dimensions (Fig. 1B). NTA further confirmed a mean particle diameter of ~ 150 nm, supporting the enrichment of exosome-like vesicles. Western blot analysis revealed that the isolated exosomes were positive for the exosomal protein markers, such as CD9 and TSG101 (Fig. 1C).

**Fig. 2:**
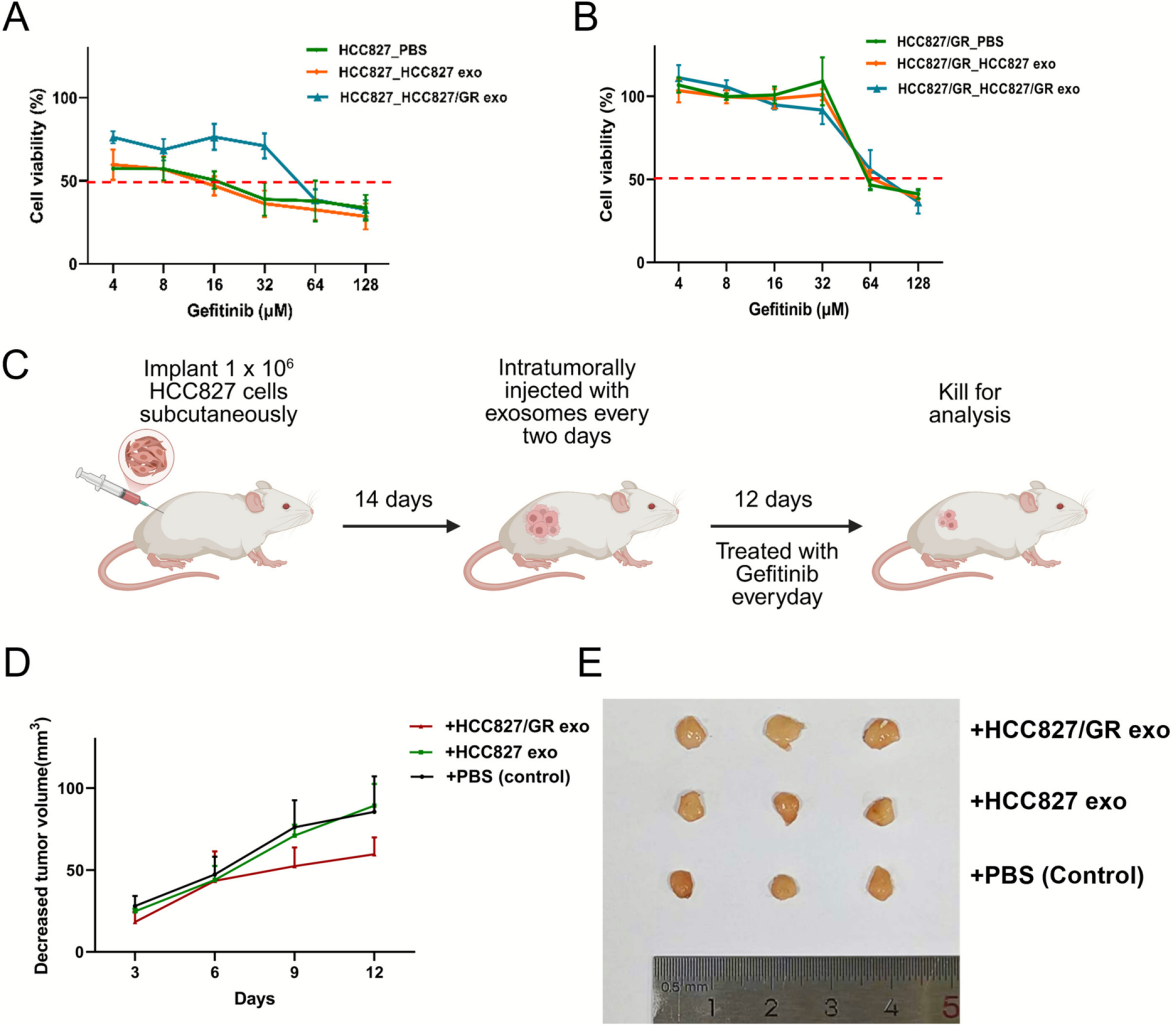
HCC827/GR cell-derived exosomes induce gefitinib resistance in HCC827 cell in in vivo and in vitro. (**A**) CCK-8 assays of HCC827 cell incubated with HCC827/GR-derived exosomes for 48 h followed by gefitinib treatment at indicated concentrations for 48 h. (**B**) CCK-8 assays of HCC827/GR cell incubated with HCC827-derived exosomes for 48 h followed by gefitinib treatment at indicated concentrations for 48 h. (**C**) Schematic diagram of in vivo animal experiments. (**D**) Subcutaneous xenograft analysis of HCC827 cells (1 × 106 cells) in nude mice with intratumoral injection of indicated exosomes upon gefitinib (5 mg/kg) treatment. The changes in tumors on different days after administration in mice were shown. The tumor shrinkage in the group treated with gefitinib combined with HCC827/GR-derived exosomes was smaller than that in the groups treated with gefitinib alone or with HCC827/GR-derived exosomes combined

We next assessed reciprocal uptake between exosomes from HCC827 and HCC827/GR cells (Fig. 1D). Fluorescence microscopy analysis confirmed that DiO-labeled exosomes derived from HCC827/GR cells were uptaken by DiI-labeled HCC827 cells after 24 h incubation, as well as exosomes from HCC827cells were uptaken by HCC827/GR cells. These results demonstrated that exosomes could be uptaken by surrounding cells and further mediated intercellular communication. Based on prior evidence that drug-resistant cell-derived exosomes can propagate chemoresistance phenotypes(Sousa et al. 2015), we examined their functional impact on the gefitinib sensitivity of HCC827 cells. Strikingly, HCC827 cells treated with HCC827/GR-exosome exhibited a two-fold elevation in gefitinib IC50 compared to control cells treated with PBS (Fig. 2A), whereas reciprocal treatment of HCC827/GR cells with HCC827-exosomes exhibited no significant changes in IC50 values (Fig. 2B). These results indicated that exosomes derived from gefitinib-resistant cells enhanced the survival of gefitinib-resistant cells with gefitinib treatment.

To further confirm that exosomes isolated from HCC827/GR cells could promote gefitinib resistance, we established a subcutaneous tumor xenograft model in athymic nude mice by implanting HCC827 cells. When the tumor volume reached 100–150 mm^3^, the mice were divided into three groups, including the control group (injected with PBS every two days), the HCC827-exosomes group (injected with exosomes derived from HCC827 cells every two days), and the HCC827/GR-exosomes group (injected with exosomes derived from HCC827/GR cells every two days). All groups were simultaneously treated with gefitinib daily, and the tumor volumes in mice were determined every three days (Fig. 2C). After gefitinib treatment, the reduction of tumor volume in the HCC827/GR-exosomes group was lower than that in the HCC827-exosomes group and control group, indicating that exosomes secreted by the HCC827/GR cells could inhibit the therapeutic efficacy of gefitinib (Figs. 2D-2E).

### HCC827/GR cells and exosomes shared hundreds of highly expressed proteins compared with the sensitive HCC827 cells

**Fig. 3:**
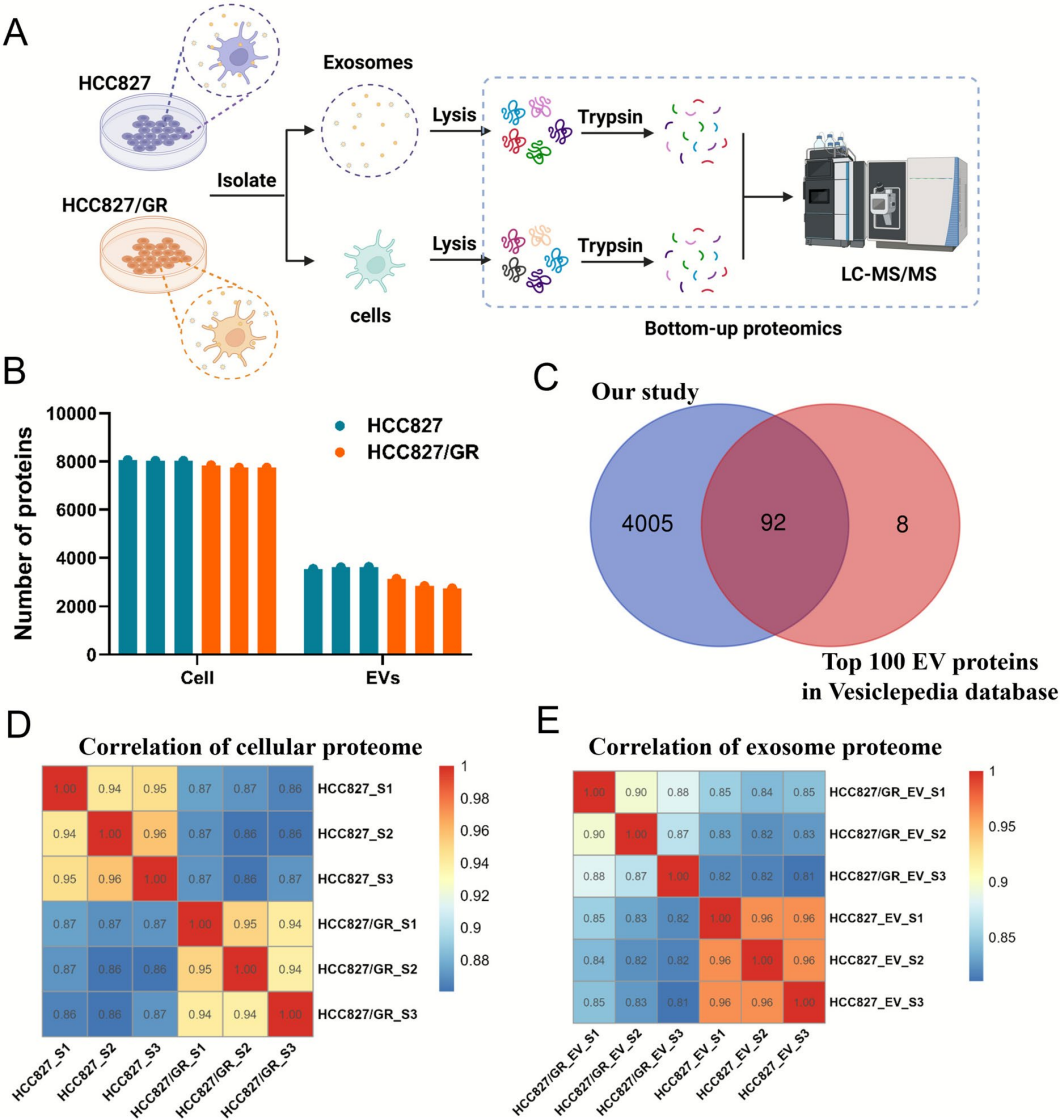
Exosome and cell proteomics analysis. (**A**) The workflow of exosomes and cell proteomics. (**B**) The number of identified cell proteome and exosome proteome. (**C**) Venn diagram of the intersection between the exosome protein set identified in this study and the Vesiclepedia Top 100 EV proteins. (**D**), (**E**) Heatmap of correlation between cellular proteome and exosome protein samples

Cell and exosomal proteomics were conducted to investigate the effects of exosome protein components on tumor microenvironments and drug resistance (Fig. 3A). A total of 8,670 proteins were identified in the cellular proteome, and 4,097 proteins were identified in the exosomal proteome (Fig. 3B, Table S1). 92 of the top 100 most reported proteins in the Vesiclepedia database were identified in our study, indicating the successful enrichment of known exosomes cargo molecules (Fig. 3C). Correlation analysis showed that HCC827/GR cells significantly differed in protein expression levels compared with HCC827 cells (Fig. 3D), as well as proteins in two corresponding exosome groups (Fig. 3E).

**Fig. 4:**
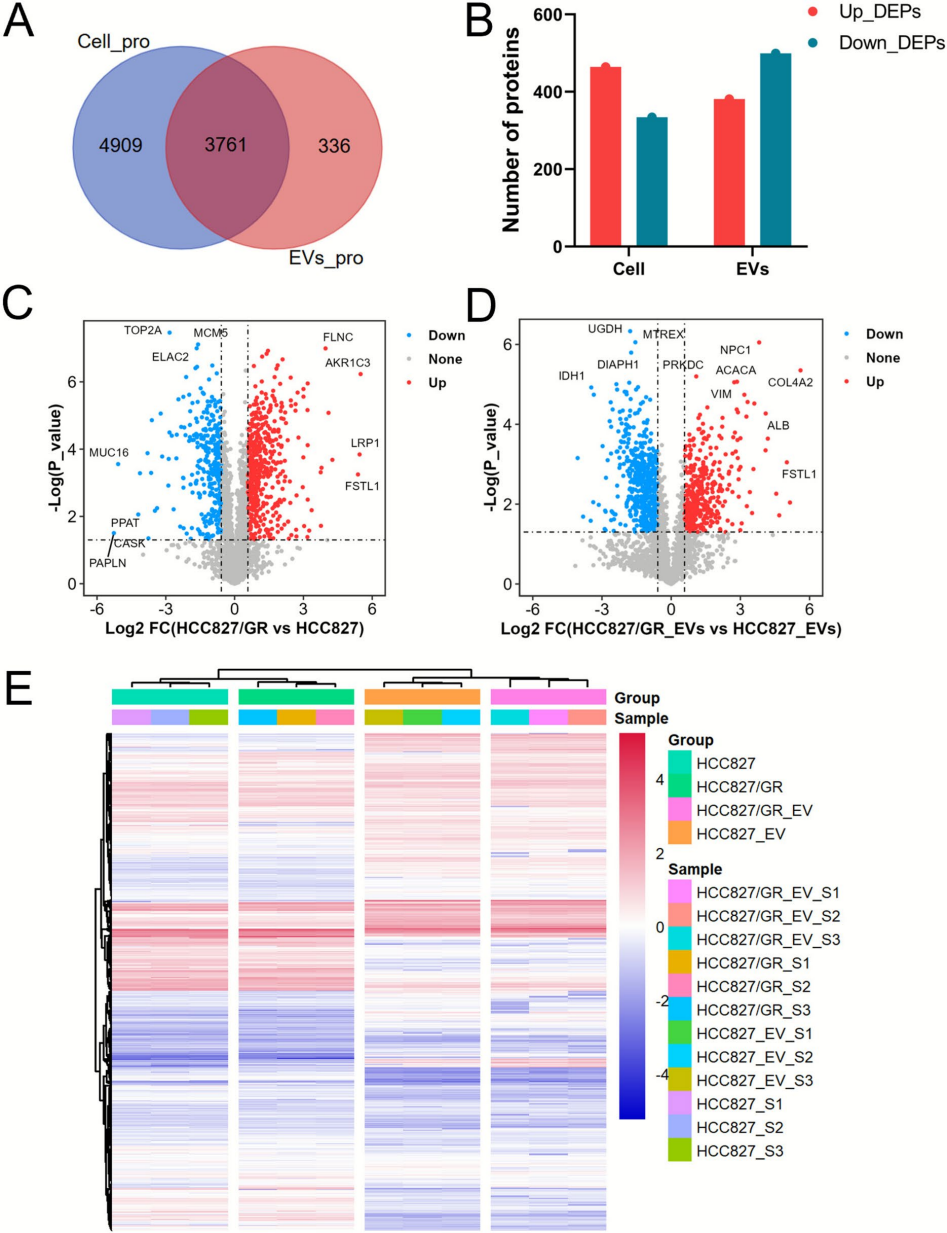
Differential analysis of cellular proteome and exosome proteome. (**A**) Venn diagram of cellular proteome and exosome proteome. (**B**) The number of differentially expressed proteins in cells and exosomes for shared proteins. (**C**), (**D**) Volcano plot of the cellular and exosome shared proteome. (**E**) Heatmap of the shared protein expression in the cellular and exosome proteome

To screen for drug resistance-related proteins, we identified 3761 proteins shared between the cellular and exosomal proteomes, representing 43.38% and 91.80% of the cellular proteins and exosome proteins, respectively (Fig. 4A). Among these shared proteins, 798 differentially expressed proteins (DEPs) were identified between cell groups (t-test p-value 0.05), with 334 proteins downregulated and 464 upregulated in HCC827/GR compared to HCC827 (Fig. 4B, 4 C). Similarly, 381 proteins were upregulated, and 499 proteins were downregulated in exosome groups (Fig. 4B, 4D). Comparing the proteomic profiles of the two groups reveals significant differences in protein expression between drug-resistant strains and their parental strains, which may be closely related to the mechanisms of drug resistance. Moreover, similar protein abundance expression profiles between cells and exosomes suggest a potential role for exosomes in the transmission of drug resistance and cell-to-cell interactions (Fig. 4E).

### Metabolic reprogramming mediates exosome-driven gefitinib resistance

**Fig. 5:**
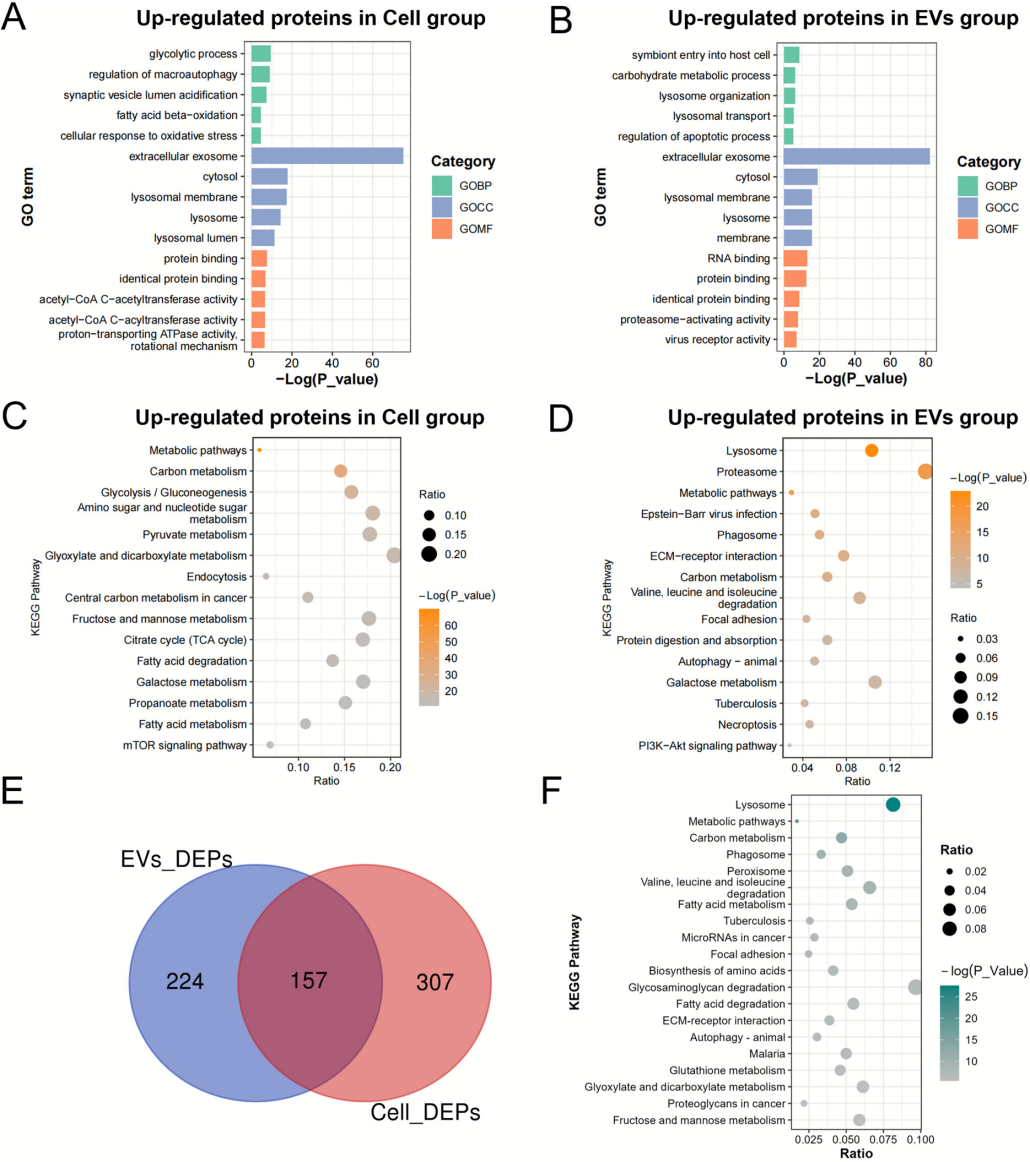
Functional enrichment analysis of upregulated shared proteins in cellular and exosomal proteomes. (**A**), (**B**) GO analysis of cellular and exosome proteome, respectively. (**C**), (**D**) KEGG analysis of cellular and exosome proteome, respectively. (**E**) Venn diagram of up-regulated shared proteins in the cellular and exosome proteome. (**F**) KEGG pathway analysis of co-up-regulated proteins in the cellular and exosome proteome

To comprehensively understand the biological functions of these differentially expressed proteins, we performed Gene Ontology analysis on the significantly upregulated proteins and downregulated proteins in both cellular and exosomal proteomes. The results indicated that upregulated proteins in the cellular proteome were closely associated with the glycolytic process, fatty acid beta-oxidation, and cellular response to oxidative stress (Fig. 5A, Table S2). This finding suggests that resistant cells may enhance these metabolic pathways to support their growth and proliferation. Conversely, downregulated proteins in the cellular proteome were predominantly enriched in DNA metabolic process, chromosomal region, and pyrophosphatase activity (Fig. S1A). Furthermore, the upregulated proteins in the exosomal proteome were related to carbohydrate metabolic process, lysosomal transport, and regulation of apoptotic process (Fig. 5B, Table S2), indicating that resistant cells can utilize exosomes to modulate intercellular signaling and the immune microenvironment, thereby enhancing their resistance to therapy. Notably, the downregulated proteins in exosomes were primarily associated with mRNA metabolic process, secretory granule lumen, and cell adhesion molecule binding (Fig. S1B).

Meanwhile, the KEGG pathway analysis results indicate that the significantly upregulated proteins in the cellular proteome are primarily associated with various metabolic pathways, including carbon metabolism, glycolysis, and fatty acid metabolism (Fig. 5C, Table S3). This finding further supports the concept that drug-resistant cells adapt to drug pressure through metabolic reprogramming. In contrast, downregulated cellular proteins were enriched in pathways governing proliferative and biosynthetic capacity, including Cell cycle, DNA replication, and Ribosome biogenesis in eukaryotes (Fig. S1C). Similarly, the significantly upregulated proteins in the exosomal proteome are related to metabolic pathways, lysosomes, proteasomes, and the PI3K-AKT signaling pathway (Fig. 5D, Table S3), reflecting the adaptability of drug-resistant cells in regulating both the intracellular and extracellular environments, as well as signal transduction. Conversely, downregulated exosomal proteins were enriched in Metabolic pathways, Spliceosome, and Protein processing in the endoplasmic reticulum (Fig. S1D). Moreover, we identified 157 commonly upregulated proteins in both datasets (Fig. 5E). These shared upregulated proteins are enriched in metabolic pathways, suggesting a potential mechanistic contribution to drug resistance (Fig. 5F, Table S4).

### Fatty acid metabolism-related enzymes promote gefitinib resistance in NSCLC

**Fig. 6:**
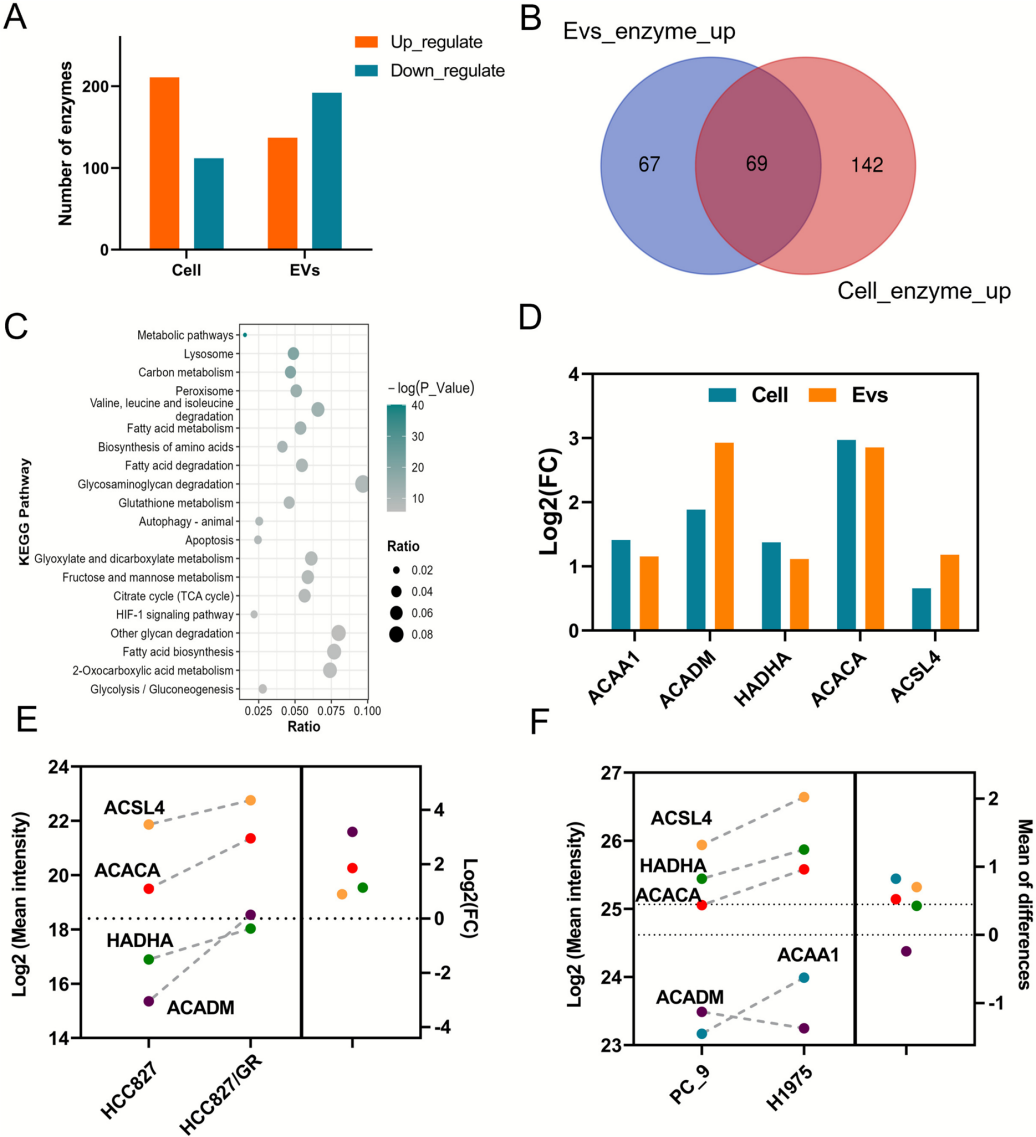
Analysis of metabolic enzymes in the cellular and exosome shared proteome. Analysis of metabolic enzymes in the cellular proteome and exosome proteome. (**A**) The number of identified enzymes and the number of differential enzymes in the cell proteome and exosome proteome (**B**) Venn diagram of the identified enzymes in the cellular proteome and exosome proteome. (**C**) KEGG analysis of the up-regulated enzymes jointly identified in the cellular proteome and exosome proteome. (**D**) Quantification of fatty acid metabolic enzymes in the cellular proteome and exosome proteome. (E) Proteomic analysis was performed on exosomes purified by differential ultracentrifugation from conditioned media HCC827 and HCC827/GR cells. (**E**) Proteomic analysis of PC9 and H1975 cells

Changes in metabolic pathways profoundly influence cellular behavior, with enzymes playing a central role in the catalytic processes within these pathways (Khan et al. 2020). We identified 882 types of enzymes in the shared proteome, of which 211 were upregulated and 112 downregulated in cells, as well as 137 upregulated and 192 downregulated enzymes in the exosomes (Fig. 6A). Notably, 69 enzymes were concordantly upregulated in both cells and exosomes (Fig. 6B). Analysis of this shared upregulated subset highlighted a pronounced enrichment in fatty acid metabolism (Fig. 6C, Table S5). Given the established role of fatty acid metabolic reprogramming in the resistance process, key enzymes, including ACC1, ACADM, HADHA, ACSL4, and ACAA1, were consistently elevated across both compartments (Fig. 6D). To exclude potential nonspecific co-precipitation and contamination by serum lipoproteins or other macromolecular impurities inherent to the PEG precipitation strategy, exosomes purified by ultracentrifugation were also tested. Similarly, ACC1, HADHA, ACSL4, and ACADM remained consistently enriched, indicating that their elevation is not a PEG-specific artifact and is methodologically robust (Fig. 6E). Moreover, to further evaluate the generalizability of this metabolic rewiring, we extended the assessment to PC9 cells bearing canonical EGFR-sensitizing mutations and H1975 cells harboring the L858R/T790M double mutation, which is representative of acquired and/or intrinsic resistance. We also found the upregulation of ACC1, ACSL4, HADHA, and ACAA1 in H1975 cells compared with PC9 cells using proteomics (Fig. 6F). Collectively, these findings suggest that the reprogramming of fatty acid metabolism constitutes an important mechanistic component of gefitinib resistance.

### ACC1 promotes gefitinib resistance in NSCLC

**Fig. 7:**
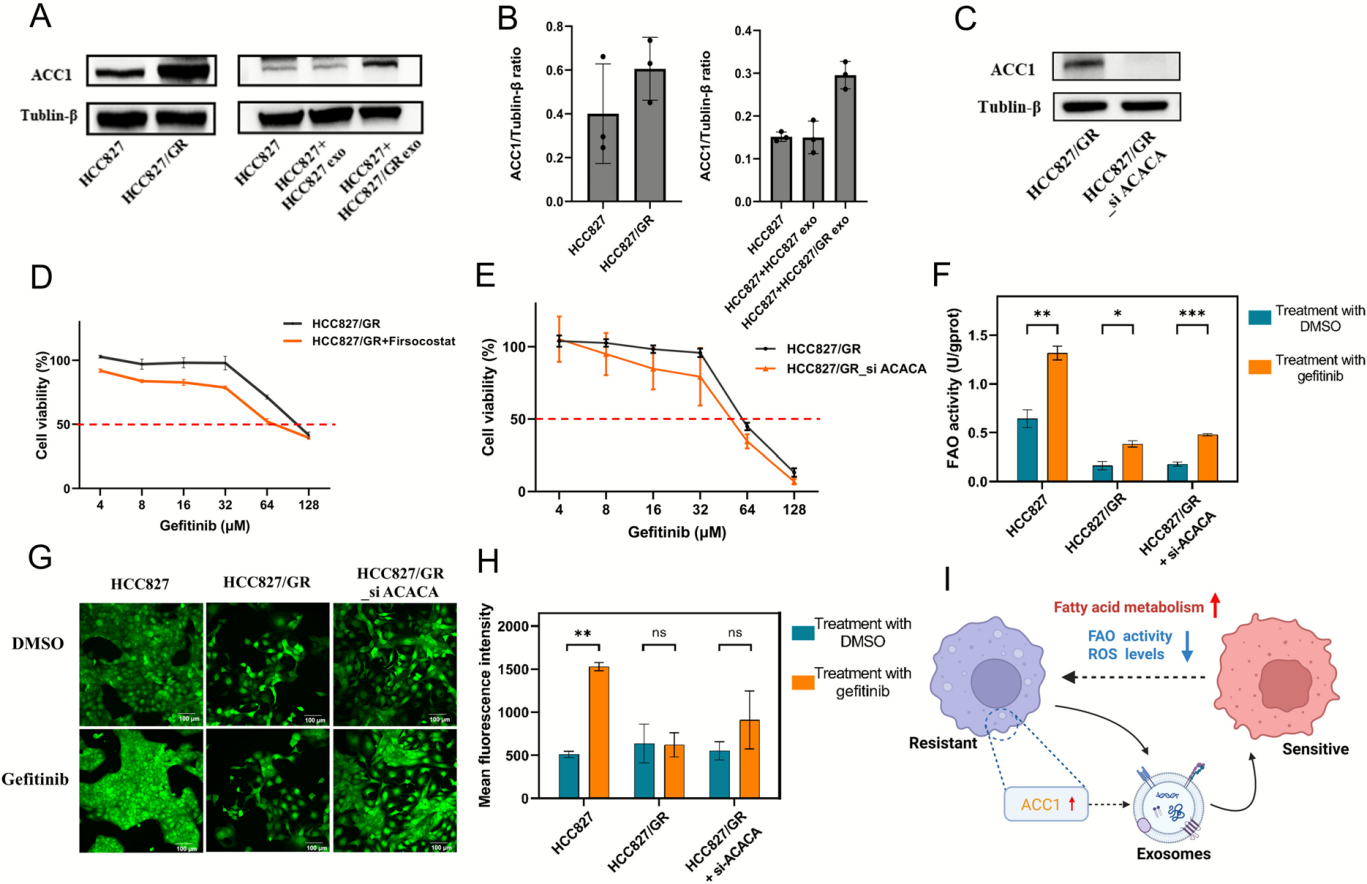
ACC1 promotes gefitinib resistance in NSCLC. (**A**) CCK-8 assay was used to detect the cell viability. HCC827/GR cells were treated with ACACA inhibitors and different concentrations of gefitinib for 48 h. (**B**) CCK-8 assay was used to detect the cell viability. HCC827/GR was transfected with si ACACA for 48 h and cells were treated with different concentrations of gefitinib. (**C**) Western blot analysis about the proteins of ACC1 in HCC827/GR cells and transfected with si ACACA. (**D**) (**E**) Western blot analysis and quantification about the proteins of ACC1 protein in HCC827 and HCC827/GR cell lines, as well as HCC827 cells incubated with exosomes from the two cell lines and PBS for 48 h. (**F**) Changes in FAO activity in different cell Lines (HCC827, HCC827/GR, HCC827/GR_si ACACA) before and after gefitinib treatment. (**G**) (**H**) Changes in ROS level in different cell Lines (HCC827, HCC827/GR, HCC827/GR_si ACACA) before and after gefitinib treatment. Scale bar, 100 μm. (I) The schematic of exosomes in the tumor microenvironment for information exchange and material transport between sensitive and resistant cell lines

The proteomics data showed that some key proteins (such as ACC1, ACSL4, and HADHA) were associated with gefitinib resistance in NSCLC cells. Among these candidates, ACC1 (encoded by ACACA), a rate-limiting enzyme of fatty acid synthesis, exhibited significant upregulation of expression with the highest fold change in both cell and exosome groups. The upregulated expression of ACC1 in HCC827/GR cell lines was also found in the results of WB analysis (Fig. 7A-7B). Furthermore, the expression of ACC1 in HCC827 cells was significantly upregulated after treatment with HCC827/GR cell-derived exosomes, whereas no difference was observed in cells treated with exosomes derived from parental HCC827 cells. This suggests that upregulated ACC1 from gefitinib-resistant cells enters surrounding cells through exosomes, thereby increasing ACC1 content in more NSCLC cells.

To determine whether ACC1 contributes to the formation of gefitinib resistance in NSCLC cells, we attenuated the activity of ACC1 in HCC827/GR cells using firsocostat (an ACC1 inhibitor) and ACC1 knockdown (Fig. 7C), respectively. The IC50 values determined by the CCK-8 assay for gefitinib in HCC827/GR cells decreased by using firsocostat, and ACC1 knockdown, indicating that ACC1 promotes the formation of gefitinib resistance in NSCLC cells (Fig. 7D-7E).

Since the homeostasis of fatty acid oxidation (FAO) and reactive oxygen species (ROS) is crucial for drug resistance, we detected FAO activity and ROS levels in 3 cell groups, including HCC827, HCC827/GR, and HCC827/GR with ACC1‑knockdown (HCC827/GR_si‑ACACA). After treatment with gefitinib, FAO activity increased the highest in HCC827 cells, the lowest in HCC827/GR cells with upregulated expression of ACC1, and was higher in ACC1‑knockdown cells than in HCC827/GR cells (Fig. 7F). Similarly, ROS levels increased in HCC827 cells and ACC1‑knockdown cells after treatment with gefitinib, and remained unchanged in HCC827/GR cells (Fig. 7G-7H). Collectively, these results suggest that ACC1 is involved in regulating intracellular FAO and ROS levels, thereby promoting the survival of NSCLC cells during gefitinib treatment (Fig. 7I).

## Discussion

Despite advancements in targeted therapies for NSCLC, such as gefitinib, resistance remains a significant barrier to effective treatment (Reungwetwattana and Dy 2013). In this study, we first demonstrated that exosomes from gefitinib-resistant HCC827/GR cells promoted the survival of sensitive HCC827 cells with gefitinib treatment, highlighting the critical role of exosome-mediated transmission of drug resistance during drug therapy. Then, proteomics of cells (HCC827 and HCC827/GR) and their secreted exosomes were conducted to investigate the role of exosomes in mediating gefitinib resistance in NSCLC. 157 proteins were identified as upregulated in both cells and exosomes when comparing the gefitinib-resistant group with the gefitinib-sensitive group. These upregulated proteins were significantly enriched in pathways related to fatty acid metabolism, which is closely associated with tumor drug resistance. This suggests that not only did gefitinib-resistant cells enhance their survival during gefitinib treatment through metabolic alterations, but also influenced the metabolism of adjacent cells by releasing exosomes.

Since a series of enzymes regulates fatty acid metabolism, investigating enzyme-level alterations is essential to pinpoint the rate-limiting steps and mechanistic nodes. A total of 882 enzymes were annotated in our proteomic data, and 69 of these were upregulated in both cells and exosomes when comparing the gefitinib-resistant group with the gefitinib-sensitive group. Among these upregulated enzymes, ACC1 is a rate-limiting enzyme in the initial process of fatty acid synthesis (Fullerton et al. 2013; Hoy et al. 2021), exhibiting the highest fold change in upregulated expression compared to other enzymes involved in fatty acid metabolism. The ACC1 was also upregulated in H1975 cells (gefitinib-resistant) compared to PC9 cells (gefitinib-sensitive), suggesting it may represent a common node in the formation of gefitinib resistance in NSCLC.

To investigate the role of ACC1 in NSCLC cells under gefitinib treatment, ACC1 was inhibited using firsocostat and knocked down by siRNA for the CCK8 assay. The reduced IC50 values from the CCK8 assay were observed in HCC827/GR cells after ACC1 inhibition or siRNA-mediated knockdown, suggesting that ACC1 promotes the formation of gefitinib resistance in NSCLC cells. Furthermore, to explore how ACC1 responds to gefitinib treatment, FAO and ROS levels were determined, and higher levels of change were observed in HCC827/GR cells after ACC1 knockdown. These results indicate ACC1 can stabilize FAO and ROS levels in NSCLC cells when gefitinib is added, thus enhancing cellular viability. Notably, ACC1 was upregulated in gefitinib-sensitive cells after the uptake of exosomes from gefitinib-resistant cells, revealing the transmission of drug resistance in NSCLC via exosomes that carry ACC1.

## Supplementary Information

Below is the link to the electronic supplementary material.

Fig. 8 Functional enrichment analysis of down-regulated shared proteins in cellular and exosomal proteomes. (A), (B) GO analysis of cellular and exosome proteome, respectively. (C), (D) KEGG analysis of cellular and exosome proteome, respectively(PNG 509 KB)

High Resolution Image (TIF 2.87 MB)

Supplementary file2 (XLSX 560 KB)

Supplementary file3 (XLSX 131 KB)

Supplementary file4 (XLSX 56 KB)

Supplementary file5 (XLSX 27 KB)

Supplementary file6 (XLSX 22 KB)

## Data Availability

The mass spectrometry proteomics data have been deposited to the ProteomeXchange Consortium via the PRIDE partner repository with the dataset identifier PXD068676.
