# Supplementary figures and images for: Exosomal proteomics reveals fatty acid metabolism linked to gefitinib resistance in non-small cell lung cancer
Source: Cell Biol Toxicol. 2025 Dec 19;42(1):3. doi: 10.1007/s10565-025-10121-8 (PMC12717232; doi:10.1007/s10565-025-10121-8)

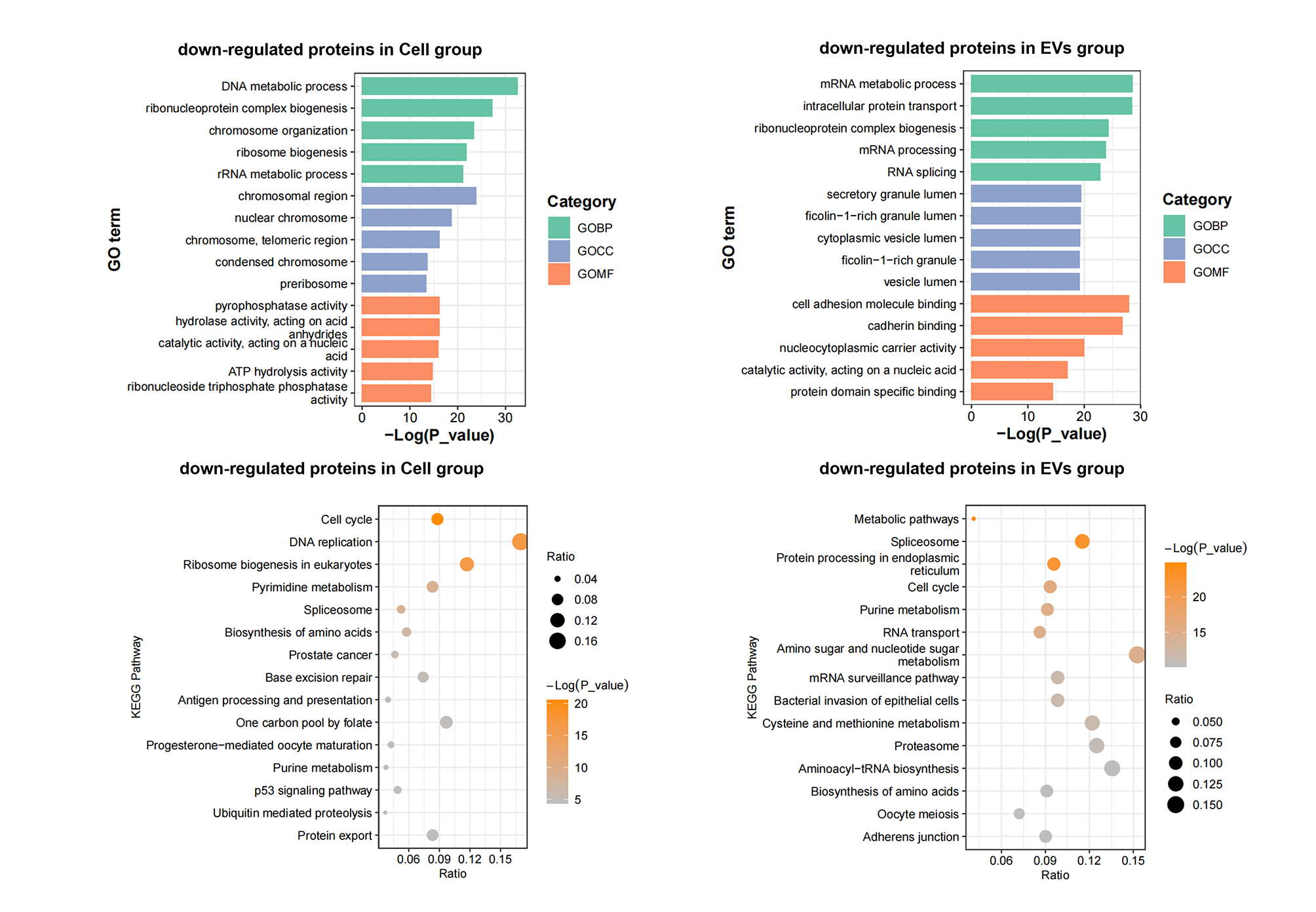

Supplement: Supplementary file 1 — Functional enrichment analysis of down-regulated shared proteins in cellular and exosomal proteomes. (A), (B) GO analysis of cellular and exosome proteome, respectively. (C), (D) KEGG analysis of cellular and exosome proteome, respectively(PNG 509 KB) [file 10565_2025_10121_Fig8_ESM.png]

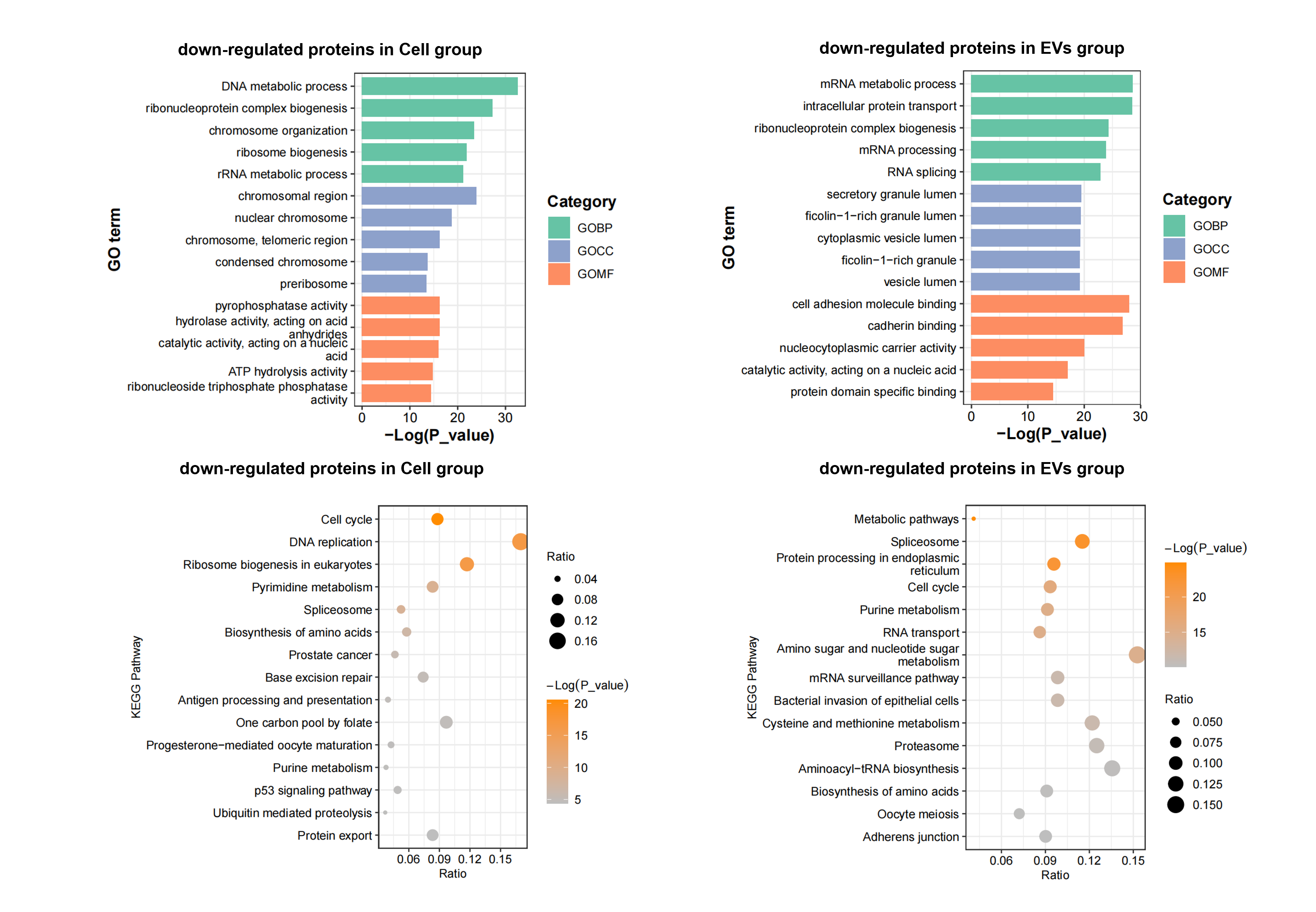

Supplement: Supplementary file 2 — High Resolution Image (TIF 2.87 MB) [file 10565_2025_10121_MOESM1_ESM.tif]
